# Supplementary material for: Endothelial cells protect Schistosoma mansoni from hydrogen peroxide-induced death
Source: PLoS Negl Trop Dis. 2026 Jan 20;20(1):e0013923. doi: 10.1371/journal.pntd.0013923 (PMC12829943; doi:10.1371/journal.pntd.0013923)
Supplement: Attachment — Submitted filename: Response to Reviewers.pdf [file pntd.0013923.s006.pdf]

FIOCRUZ

September 15th, 2025

To the Editor-in-Chief
PLOS Neglected Tropical Diseases

Subject: Submission of Manuscript: " *Endothelial cells protect Schistosoma mansoni from hydrogen peroxide-induced death* "

Dear Editor,

Thank you for your guidance. We have carefully revised the manuscript and submission files to address all issues raised:

1. All trademark/copyright symbols have been removed from the text.
2. A complete list of Supporting Information legends (S1–S4 Tables) was added after the References section.
3. We have initiated the submission process for data deposition in the Arca Dados repository (https://arcadados.fiocruz.br/). The dataset will be fully available before article acceptance, in compliance with PLOS data sharing policy.
4. A dedicated "Financial Disclosure" section was created, including a statement on the funders' role: *The funders had no role in study design, data collection and analysis, decision to publish, or preparation of the manuscript.*
5. Funders' names and grant numbers were harmonized between the manuscript and the Funding Information tab to ensure exact correspondence.

We trust the manuscript is now in compliance and look forward to the next steps in the editorial process.

Sincerely,

Ricardo Riccio Oliveira**, PhD**
On behalf of all authors
Fiocruz
ricardo.riccio@fiocruz.br
