# Supplementary material for: Endothelial cells protect Schistosoma mansoni from hydrogen peroxide-induced death
Source: PLoS Negl Trop Dis. 2026 Jan 20;20(1):e0013923. doi: 10.1371/journal.pntd.0013923 (PMC12829943; doi:10.1371/journal.pntd.0013923)
Supplement: Attachment — Submitted filename: Response to Editor and Reviewers.docx [file pntd.0013923.s007.docx]

**Response to Editors and Reviewers**

**Manuscript ID:** PNTD-D-25-01246R1

**Title:** Endothelial cells protect *Schistosoma mansoni* from hydrogen peroxide-induced death

Dear Editors,

We sincerely thank you for the careful evaluation of our manuscript and for the opportunity to submit a revised version. We deeply appreciate the constructive recommendations provided in the “Journal Requirements” section, which we have addressed point by point below.

Before presenting these responses, however, we would like to respectfully request clarification regarding the peer-review reports. As detailed later in this letter, the comments attributed to Reviewer #2 appear to refer to a population-based epidemiological study in malaria, not to our in vitro experimental work on *Schistosoma mansoni* and endothelial cells. Additionally, Reviewer #1 appears as “No Response”. Because these issues significantly affect our ability to respond adequately to the scientific evaluation, we kindly ask for confirmation before proceeding with the full scientific rebuttal.

Below we present our detailed responses to all editorial requirements and our request for clarification regarding the reviewer reports.

**A. Response to the Editors – Journal Requirements**

**1. Duplicate Files in the Submission System:** *We note that the main files of the manuscript are duplicated on your submission. Please remove any unnecessary or old files ("SouzaBOL_Manuscriptv1.pdf) from your revision, and make sure that only those relevant to the current version of the manuscript are included.*

We appreciate the Editors’ careful review of the submission materials.
We removed all unnecessary and outdated files from the submission system, including the file “SouzaBOL_Manuscriptv1.pdf.” Only the files corresponding to the revised and current version of the manuscript were retained.

**2. Removal of Trademark and Copyright Symbols:** *We do not publish any copyright or trademark symbols that usually accompany proprietary names, eg ©, ®, or TM (e.g. next to drug or reagent names). Therefore please remove all instances of trademark/copyright symbols throughout the text, including:- TM on pages: 7, and 8.*

We thank the Editors for highlighting this stylistic requirement.
We carefully reviewed the entire manuscript and removed all trademark and copyright symbols (e.g., TM, ®, ©), including those identified on pages 7 and 8. The full text was adjusted to comply with PLOS NTD style guidelines.

**3. Addition of Supporting Information Legends:** *We have noticed that you have uploaded Supporting Information files, but you have not included a list of legends. Please add a full list of legends for your Supporting Information files after the references list.*

We acknowledge and appreciate the reminder.
We added a complete list of legends for all Supporting Information files. This section was inserted immediately after the References list, as required.

**4. Data Availability Requirements:** *Thank you for stating "All relevant data will be made available upon publication through the Arca Dados platform, the official data repository of Fiocruz, at https://arcadados.fiocruz.br/." Please note that, though access restrictions are acceptable now, your entire minimal dataset will need to be made freely accessible if your manuscript is accepted for publication. This policy applies to all data except where public deposition would breach compliance with the protocol approved by your research ethics board.*

We fully agree with PLOS data availability policies.
We reaffirmed adherence to PLOS data-sharing policies and revised the Data Availability Statement accordingly. We clarified that the complete minimal dataset will be made publicly accessible in the Arca Dados repository upon acceptance, with no restrictions other than those required for ethical compliance.

**5. Revision of the Financial Disclosure Statement:** *Please amend your detailed Financial Disclosure statement. This is published with the article. It must therefore be completed in full sentences and contain the exact wording you wish to be published.*

*1) State what role the funders took in the study. If the funders had no role in your study, please state: "The funders had no role in study design, data collection and analysis, decision to publish, or preparation of the manuscript.".*

We thank the Editors for this guidance.

We fully rewrote the Financial Disclosure section using complete sentences and included the exact wording requested by the Editors. The statement now reads:

“The funders had no role in study design, data collection and analysis, decision to publish, or preparation of the manuscript.”

This information is now presented precisely as it should appear upon publication.

**B. Response to Reviewers**

Before addressing specific comments, we respectfully request clarification regarding the peer-review reports received.

The comments attributed to **Reviewer #2** refer extensively to a *population*, *sample size*, *epidemiological characteristics*, *malaria control*, and *surveillance systems*, as well as ethical considerations related to *human participants*. However, our manuscript presents an **in vitro experimental study** involving *Schistosoma mansoni* and **endothelial cells**, with no human population, no epidemiological component, and no sample-size calculations applicable to population-based research.

Additionally, **Reviewer #1** appears as “No response”.

Therefore, before preparing point-by-point revisions to reviewer comments, we kindly ask the editorial team to confirm:

1. Whether the comments provided for Reviewer #2 correspond to our manuscript or were inadvertently associated with our submission.
2. Whether Reviewer #1 intended to submit a blank review or whether their comments are missing due to a technical issue.

We will be pleased to revise the manuscript according to any appropriate scientific feedback once the correct reviews are provided.

Once again, we thank the Editors for their thoughtful guidance and the opportunity to revise our manuscript.

We look forward to your guidance regarding the reviewer reports so that we may finalize our responses.

Sincerely,

**Ricardo Riccio Oliveira**
On behalf of all co-authors
